# Elbow Damage Identification Technique Based on Sparse Inversion Image Reconstruction

**DOI:** 10.3390/ma13071786

**Published:** 2020-04-10

**Authors:** Yu Wang, Xueyi Li

**Affiliations:** School of Mechanical Engineering, Xi’an Jiaotong University, Xi’an 710049, China; lxy_engineer@stu.xjtu.edu.cn

**Keywords:** ray tracing, tomographic of elbow, sparse inversion, elbow damage identification

## Abstract

Continuous monitoring for defects in oil and gas pipelines is important for leakage prevention. This paper proposes a new kind of pipe elbow damage identification technique, which consists of three processes. First, piezoelectric sensors evenly arranged along the circumference of the pipeline in the turn generated ultrasonic guided wave signals in the elbow. Then, the wavefront flight time at each grid node in the known sound field were computed using the fast-marching algorithm. Finally, an elbow wall thickness map reconstruction technique based on the sparse inversion method was proposed to identify elbow defects. Compared with the traditional elbow defect identification technology, this technology can not only detect the existence of the defect but also accurately locate the defect position.

## 1. Introduction

Industrial pipelines are widely used for many applications and are the lifelines of cities worldwide. To avoid unexpected leaks or ruptures, identification of pipeline damage is of major importance [1,2]. Among potential pipeline leakage locations, pipe elbows are important but difficult to detect due to their characteristics of easy accumulation, strong impact, and complex structure [3].

Sensing technologies are important for locating defects. Traditional ultrasonic detection technology scans all points on the surface of interest and detects defects by ultrasonic propagation time and wave velocity. This method requires a high time cost and must have access to all surfaces of interest [4]. In recent years, a new member of the ultrasonic family, ultrasonic guided wave detection technology, has been developed and, in addition to the advantages of general ultrasonic detection, can carry out long-distance transmission along the detected structure [5]. Structural defects can be realized with one detection point, so the efficiency is high [6]. In addition, the ultrasonic sound field guided wave spreads throughout the whole wall thickness of the detected component, and, thus, the internal and external defects of the structure can be detected simultaneously [7]. Ultrasonic guided wave tomography, which is an important quantitative imaging methods, also removes the limits of sensors that need access to all points in the areas of interest [8]. 

Ultrasonic guided wave tomography [9,10,11] comes from the computed tomography (CT) technology used in medical fields. Sensors are arranged around the detection area, and the projected data are obtained by scanning the detection area according to the mode of "one sends while others receive" [12]. Then, the physical damage is quantified and reconstructed through the inversion algorithm, and the image pixels are obtained [13,14]. Jansen et al. used lamb wave tomography and C-scans technology to detect the polymer composite plate respectively, and realized the damage identification of matrix cracking, delamination and fiber failure [15]. Pei et al. demonstrated lamb wave tomographic techniques and presented the results of defect measurements of an aluminum plate [16]. Combining filtered back-projection tomography with laser ultrasound, Nagata et al. used line-focused pulse laser to generate lamb waves and took the propagation attenuation of guided waves in the defect area as the imaging parameter to get tomographic imaging of through-holes defects in thin aluminum plates [17]. Hinders et al. demonstrated that cross projection is an adaptable method for guided wave tomography and used for scanning structures of any geometric shape [18] and a multi-mode ultrasonic guided wave tomography method was also proposed to identify different types of damage in an aluminum plate [19]. With the combination of cross-hole tomography and ultrasonic guided wave technology, Leonard et al. realized the ultrasonic guided wave tomography detection for pipeline defects [20] and then used ultrasonic signals that travel with various helical paths to achieve tomographic reconstruction for pipes [21]. Rao et al. verified that the full-waveform inversion algorithm has a good reconstruction accuracy for the defects of plate-like structures, and proved that the full-waveform inversion algorithm has a better accuracy for multiple defects than compared to the Born approximation algorithm [22]. Based on first order of the Born approximation algorithm and Mindlin plate theory, Wang et al. derived analytical solutions in diffraction wave fields and a diffraction tomography technology to quantify defect in plate-like structures [23]. Huthwaite et al. studied high-precision guided wave tomography and compared the inversion methods of guided wave tomography in detail [24,25,26]. Based on mathematical statistics, Gao H et al. proposed a probabilistic damage reconstruction algorithm (Reconstruction Algorithm for Probabilistic Inspection of Defects, RAPID), which uses signal difference coefficients as tomographic imaging parameters to describe the probability of damage caused by different scanning paths in the sensor network [27]. Zhang et al. developed the defect tomography technology of aluminum plate, and proved that the defect size reconstructed by the bending ray algebraic reconstruction technology is closer to that of linear ray algebraic reconstruction [28]. Information on the guided wave signal that circles the pipe several times before it reaches the receiving array was extracted by Simonetti; the results of wall thickness construction were as accurate as those of ultrasonic thickness measurements at single point [29,30]. Gao et al. used probability reconstruction algorithm and hyperbolic algorithm to obtain the reconstructed image of the defect with cladding layer, respectively, and showed that the probability reconstruction algorithm has a faster imaging speed [31].

The resolution of ultrasonic guided wave tomography depends mainly on the scanning density of the sensor network in the detection area. The higher the density, the more complete the projected data collected will be. However, this will result in that the imaging efficiency is greatly reduced [27]. Therefore, sparsity methods were used in ultrasonic wave tomography to improve the imaging efficiency. Bai et al. introduced a new algorithm for reconstructing the defect image from sparse measurements in plate with Huber regularization [32]. Li et al. studied the defect scattering distribution for guided waves signals and designed reasonable layout of sparse sensors for good reconstruction quality [33]. The process of tomography can be regarded as an inversion problem in which the detected structure state can be obtained using the signal monitored by ultrasonic transducers [34,35]. The detected structure has prior information on the physical characteristic of the undamaged structure. Based on this, Brath et al. proposed a sparse model that the slowness difference between defective and non-defective sound fields was used as a reconstruction parameter [36]. Then, a sparse LASSO regression model built by Gao et al. was used to avoid artifacts before the reconstruction of the defect in plate-like materials [37].

This paper proposes an image reconstruction technique that uses a sparse inversion method for elbow damage identification and thus ameliorated the efficiency of guided wave tomography for elbows. Before reconstruction, a second-order difference of flight time was derived to approximate its gradient to offer more sound field information. Then, a threshold setting for sparse inversion was proposed. Only the sound field points where the transformation of guided wave velocity was larger than the set threshold participated in the solution process. The guided wave velocity was a function of wall thickness when the central frequency was fixed [38]. The connection between the wall thickness and the wave velocity was then established. According to the minimum resolution of the change of wall thickness, the threshold of the wave velocity in the inversion process could be set.

The rest of this paper is organized as follows: A 2-D forward acoustic model was built to simplify the imaging process, and a ray tracing technique was used to associate the signal characteristics with the elbow physical model in Section 2. A sparse inversion technique based on lamb wave dispersion curves to reconstruct the elbow wall thickness map was proposed in Section 3. Experimental validation of the sparse inversion method proposed was provided in Section 4 where the performance of identifying elbow defect in extrados was studied. Conclusions are presented in Section 5.

## 2. Forward Model and Ray Tracing

### 2.1. 2-D Forward Model

The model based on a 3-D electrokinetic equation could not be solved easily [38]. A simplified forward model was built by unwrapping the pipes to a plate with the assumption that the wall thickness of the pipe was small relative to its diameter [39]. According to tomographic imaging principle and Fermat’s theorem, the approach introduced in [40] established the heterogeneous anisotropic sound field model to transform the dual mapping of the elbow 3-D model to the 2-D model in physical structure as well acoustic characteristics.

**Figure 1:**
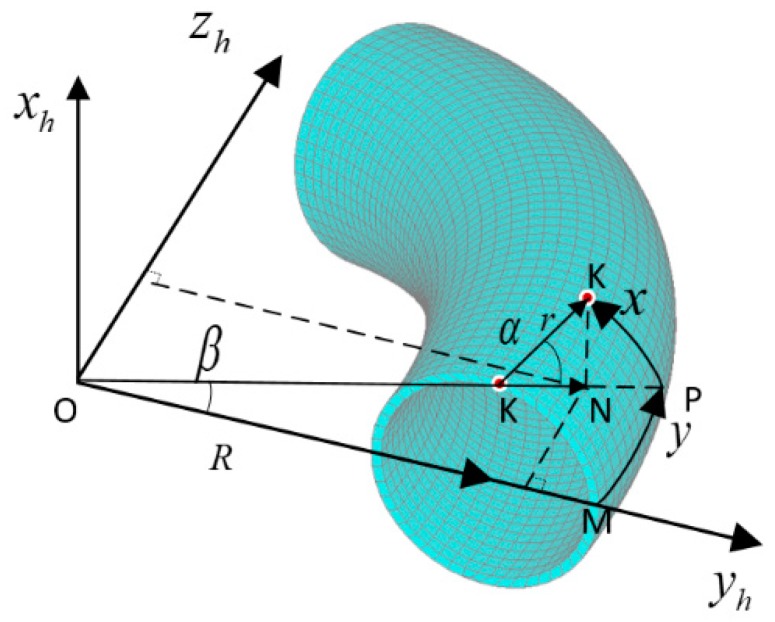
Coordinate system used to simplify forward model.

The outer ridge of the bend was the y axis system, and the direction perpendicular to the ring was the x axis in the two-dimensional coordinate system. The specific structure is shown in Figure 1.

In Figure 1, two key angles are defined to transform the three-dimensional geometric model to the two-dimensional geometric model. Their expressions are:

(1) $$\alpha =\frac{x}{r},\beta =\frac{y}{R+r}$$

where r is the outer radius and *R* is the bending radius of the elbow.

Then, a geometric transformation that uses two-dimensional coordinates {x,y} to express three-dimensional space coordinates $\left\{x_{h},y_{h},z_{h}\right\}$ was conducted, namely:

(2) $$\{\begin{array}{c} x_{h}=r\sin\alpha =r\sin\frac{x}{r}\\ y_{h}=(R+r\cos\alpha )\cos\beta =(R+r\cos\frac{x}{r})\cos\frac{y}{R+r}\\ z_{h}=(R+r\cos\alpha )\sin\beta =(R+r\cos\frac{x}{r})\sin\frac{y}{R+r} \end{array}$$

**Figure 2:**
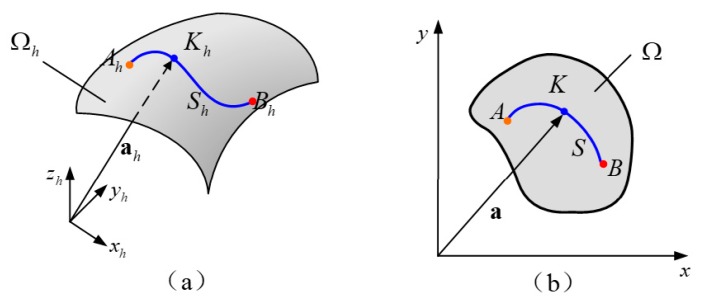
Ultrasonic guided wave propagation path in different coordinate systems of (**a**) the 3-D surface of elbow and (**b**) the 2-D mapped surface.

The three-dimensional propagation path $S_{h}$ of the ultrasonic guided wave from point $A_{h}$ to point $B_{h}$ is shown on the three-dimensional elbow surface $\Omega_{h}$ shown in Figure 2a. The two-dimensional plane Ω shown in Figure 2b, represented the ultrasonic guided wave propagation path from point A to point B in the two-dimensional mapping.

By introducing the anisotropic sound velocity factor θ, which is the angle between the propagation direction of the guided wave and the x axis in the two-dimensional model, the 2-D sound field v(a,θ) was determined to be:

(3) $$v(a,\theta )=\frac{c_{h}[a_{h}]}{\sqrt{\cos^{2}\theta +\alpha^{2}(a)\sin^{2}\theta }}$$

where α(a) is the sound field factor, written as:

(4) $$\alpha (a)=\frac{R+r\cos(x/r)}{R+r}$$

**Figure 3:**
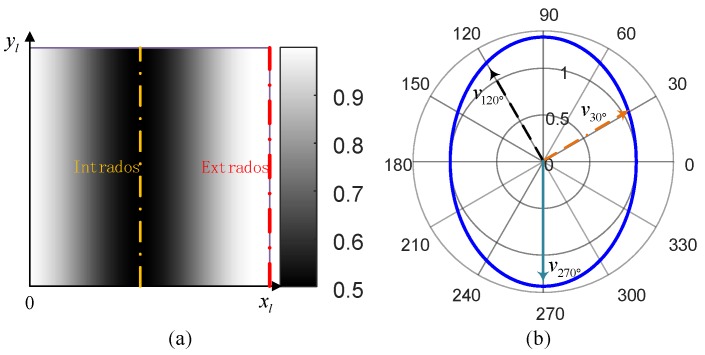
Sound field model analyses: (**a**) non-uniformity analysis and (**b**) anisotropic analysis.

From analysis of the sound field factor, the 2-D sound field had the following characteristics:

(1) The sound field factor changed with the change of x shown in Figure 3a, which indicated that the propagation speed of guided wave as different at different positions. Thus, the sound field was heterogeneous.
(2) $\alpha \in [\frac{R-r}{R+r},1]$, which indicated that, in the outer arc of the bend, the sound field could be seen as isotropic while in other areas the sound field was anisotropic. The anisotropy of the sound field expressed in Figure 3b could be more intuitive, and the guided wave had the maximum propagation velocity in the y-direction at the inner arc of the pipeline.

### 2.2. Ray Tracing Method

To associate the signal characteristics with the elbow physical model, further evolution of the proposed forward model was required. Based on the constructed two-dimensional heterogeneous anisotropic sound velocity field model, the correlation between sound field information and flight time characteristics of guided wave signals were established.

Ultrasonic guided wave flight time was an important property parameter of the guided wave. The geophysical method for solving the forward modeling problem of a complicated geological model was a complicated physical model discretization to form a series of sound field unit grid [41,42,43]. Thus, the parameters of the acoustic field model were simplified to analyze the propagation characteristics of the guided wave.

Vidale proposed a ray tracing method based on solving the Eikonal equation, named the finite difference method in 1988 [44]. Later research improved the finite difference method and proposed a stable and accurate wavefront flight time calculation method called the Fast Marching Method (FMM) [45,46,47].

The entropy used to solve the equation satisfied the upward region, written as:

(5) $$\max{\left(D_{{}_{i,j}}^{-x}t,-D_{{}_{i,j}}^{x}t,0\right)}^{2}+\frac{\max{\left(D_{{}_{i,j}}^{-y}t,-D_{{}_{i,j}}^{y}t,0\right)}^{2}}{\alpha^{2}}=\frac{1}{v_{{}_{i,j}}^{2}}$$

where $D_{{}_{i,j}}^{-x}$ is the backward difference along the direction, $D_{{}_{i,j}}^{x}$ represents the forward difference along the direction, and the other two difference forms are the same, $v_{{}_{i,j}}^{}$ is the guided wave velocity at position (i,j).

**Figure 4:**
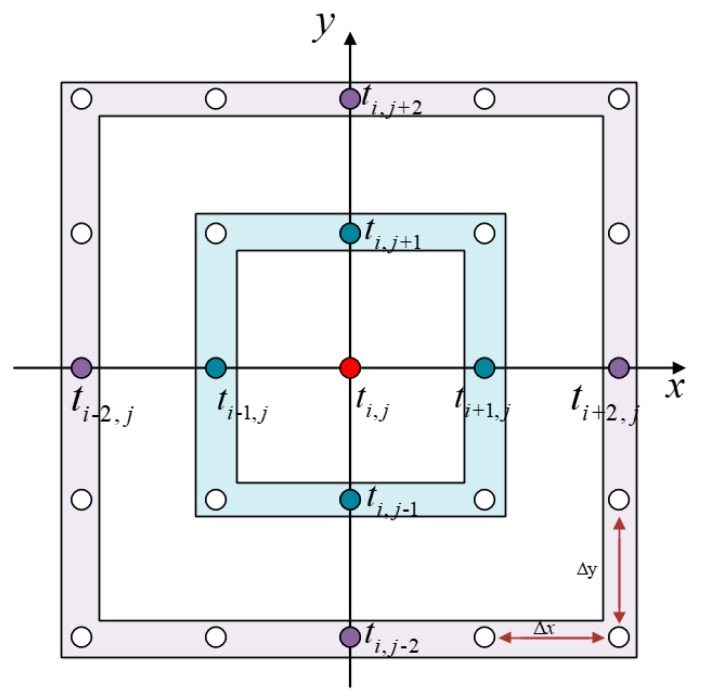
The point distribution for the calculation of the guided wave flight time. At most eight points of travel time information is required in second-order difference form.

The second-order difference form was used to approximate the gradient of flight time and written as:

(6) $$\{\begin{array}{l} D_{{}_{i,j}}^{-x}=\frac{3t_{i,j}-4t_{i-1,j}+t_{i-2,j}}{2\Delta x}\\ D_{{}_{i,j}}^{x}=\frac{3t_{i,j}-4t_{i+1,j}+t_{i+2,j}}{2\Delta x}\\ D_{{}_{i,j}}^{-y}=\frac{3t_{i,j}-4t_{i,j-1}+t_{i,j-2}}{2\Delta y}\\ D_{{}_{i,j}}^{y}=\frac{3t_{i,j}-4t_{i,j+1}+t_{i,j+2}}{2\Delta y} \end{array}$$

where $t_{i,j}$ is an unknown point in the sound field shown in Figure 4. The second order difference operator was substituted into the formula to obtain the ultrasonic guided wave travel time $t_{i,j}$.

## 3. Image Reconstruction

### 3.1. Sparse Inversion Image Reconstruction

Different from the image reconstruction in the field of geological survey, the accuracy of the elbow defect identification required higher resolution, which led to a long inversion calculation and low efficiency. In this section, a sparse inversion image reconstruction technology was developed based on the sparsity of defect distribution so as to improve the efficiency of the algorithm for solving the defect image reconstruction of the elbow.

The purpose of image reconstruction for elbow was to obtain the pipe wall thickness loss image based on the flight time matrix T. When the guided wave velocity matrix of the elbow sound field was determined, the pipe wall thickness information could be obtained according to the dispersion curve.

Consider the nonlinear inversion problem:

(7) T=F(v)

where T is the fight time matrix, v is the guided wave velocity matrix of the elbow sound field, and F is the forward operator obtained by ray tracing.

When defining the guided wave velocity change matrix $\Delta t=t-t^{\prime }$, where $v^{\prime }$ is the guided wave velocity matrix of elbow without defect, the inversion problem is reconstructed as:

(8) T=F(v−Δv)=A(Δv)

where A is the modified forward operator according to the guided wave velocity change matrix Δv.

Considering the existence of the error, the equation was written as:

(9) T=A(Δv)+ε

Therefore, the optimization objective of the ultimate inversion problem is:

(10) $$\underset{\varepsilon }{\min}{\left\|\varepsilon \right\|}_{2}$$

And the loss function was defined as:

(11) $$M(\Delta v)={\left\|\varepsilon \right\|}_{2}={\left\|T-A(\Delta v)\right\|}_{2}$$

In this paper, image reconstruction of pipe elbow was based on the steepest descent method. [48] The iterative format in step n was as follows:

(12) $$\Delta v^{n+1}=\Delta v^{n}-k^{n}\delta^{n}$$

where k is the step size, δ is the descent direction, $\delta^{n}=\nabla M(\Delta v)$.

**Figure 5:**
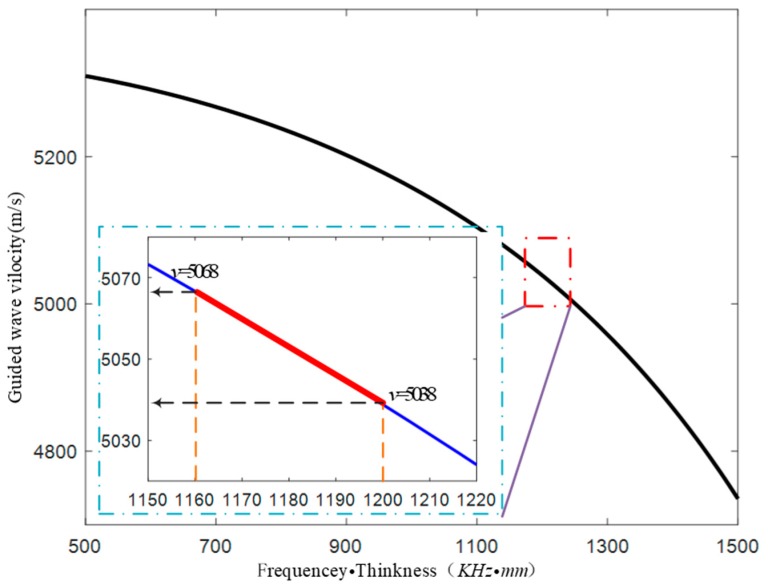
The setting of sound field threshold. Based on the dispersion curve, it can be deduced that the guided wave velocity boundary is 5068 corresponding to the defect recognition resolution of 0.1 mm.

Based on the sparsity characteristics of defect distribution, sparse inversion image reconstruction was carried out. The setting process of the inversion threshold was the core of the sparse inversion algorithm. As shown in the Figure 5, it was assumed that the guide wave was excited at the center frequency of 200 KHz, and the elbow wall thickness was 6 mm. To achieve the defect recognition resolution of 0.1 mm, the threshold for change of guided wave velocity was calculated as 30 m/s according to the dispersion curve (20#, elastic modulus = 210 GPa, Poisson ratio = 0.3, density = 7830 kg/m^3^).

**Figure 6:**
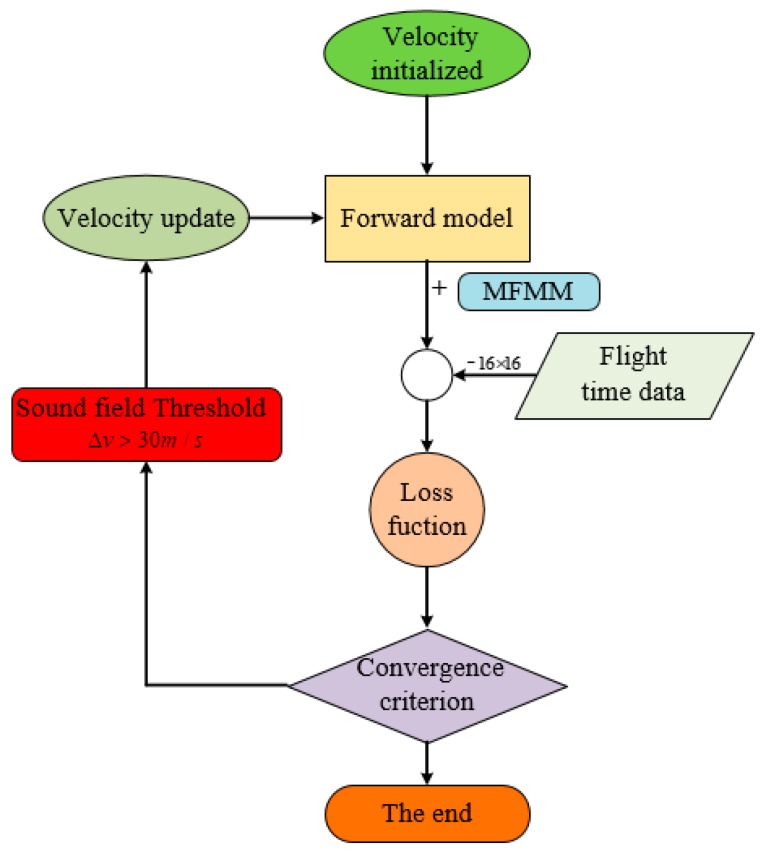
The flowchart of sparse inversion image reconstruction.

The flowchart of sparse inversion image reconstruction is shown in Figure 6. The sound field model is initialized to the defect-free bend sound field. The travel time information of the guided wave in the sound field is obtained by the forward model and the loss function is established by compared with the flight time data of experiment. The defect reconstruction image is output until the convergence condition is reached. In the iterative process, the threshold of sound field was introduced at the thickness resolution of 0.1 mm, and the forward calculation was carried out after the sound field was updated.

### 3.2. Simulation Verification

**Figure 7:**
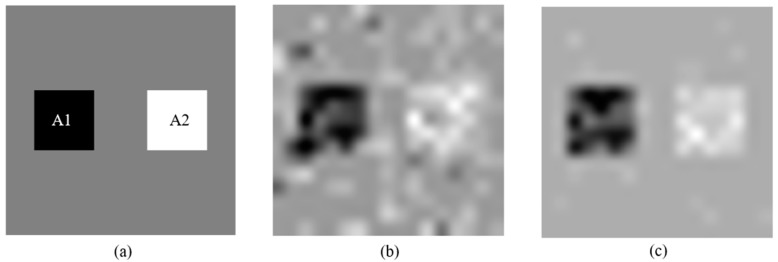
(**a**) Initial sound field model; (**b**) reconstruction results with the sound field threshold set as 0, which means non-sparse inversion method was used; and (**c**) reconstruction results with the sound field threshold set as 100.

Based on the two-dimensional anisotropic sound velocity field model and ray tracing method in Section 2, the simulated sound field model was established to test and evaluate the effect of sparse inversion image reconstruction technology shown in Figure 7a. The model has two distorted velocity regions: high speed distortion region A1 (wave velocity 4500 m/s) and low speed distortion region A2 (wave velocity 3500 m/s). The model parameters are shown in Table 1.

In the reconstruction model, the background velocity was taken as initial global wave velocity. The sound field threshold is set to 0 (non-sparse inversion) and 100 (sparse inversion). The reconstruction results were shown in Figure 7b,c, respectively.

We can easily distinguish two velocity distortion regions in the reconstructed sound field in Figure 7c reconstructed by sparse inversion method. Due to the introduction of sound field threshold, the unknown quantity in reconstruction process was reduce and the noise was also reduced. The reconstruction result shown in Figure 7c retains more accurate characteristic of the sound field compared with reconstruction result shown in Figure 7b.

## 4. Experiment and Results

### 4.1. Establishment of Ultrasonic Guided Wave Experimental System

**Figure 8:**
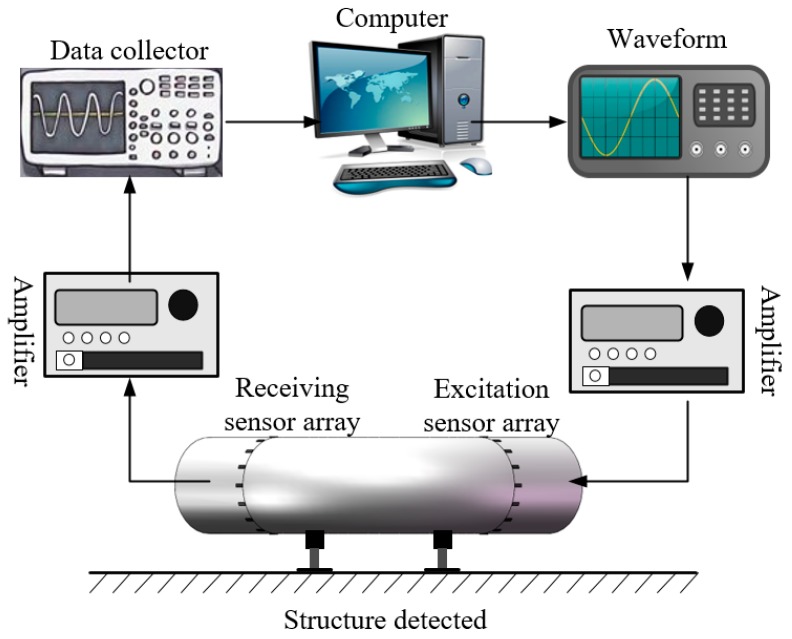
The diagram of ultrasonic guided wave detection scheme.

According to its principles, ultrasonic guided wave detection is an active detection technique. Therefore, the ultrasonic guided wave detection system should be composed of a signal excitation unit and a signal acquisition unit, as shown in Figure 8.

**Figure 9:**
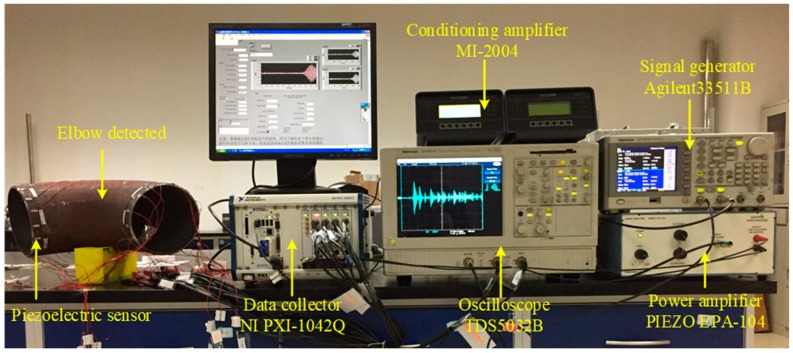
Ultrasonic guided wave detection experimental platform.

In the ultrasonic guided wave detection scheme diagram shown in the Figure 6, the signal excitation unit was composed of a waveform generator, an amplifier and an excitation sensor, while the signal acquisition unit was composed of a data collector, an amplifier and a receiving sensor. During the detection process, the signal generator generated the characteristic wave detection signal, which was amplified by the amplifier. Then, the excitation sensor converted the voltage signal to the mechanical vibration signal to stimulate the ultrasonic guided wave in the elbow. The guided wave signal with structure characteristic information was converted to a voltage signal that was monitored through the receiving sensor. After being amplified by the signal amplifier, it was collected and stored by the data collector and finally analyzed and processed by the computer. According to the diagram, an ultrasonic guided wave detection experimental platform as shown in Figure 9 was built.

**Figure 10:**
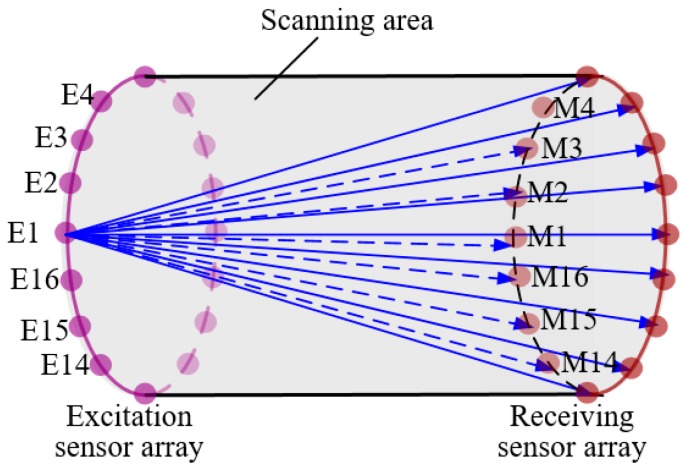
Cross-hole scanning structure.

According to the cross-hole scanning principle [19], during the detection process, only one sensor in the excitation sensor array loop was used as the excitation source to generate the detection guide wave. Simultaneously, the receiving sensor collected the detection signal. As shown in Figure 10, the detection signals generated by excitation sensor E1 were received by the M1 to M16 sensors in the receiving sensor array ring. Then, the excitation sensor was switched to the E2 excitation sensor, and the same data acquisition process was conducted until the sensors in the excitation array ring were all scanned.

**Figure 11:**
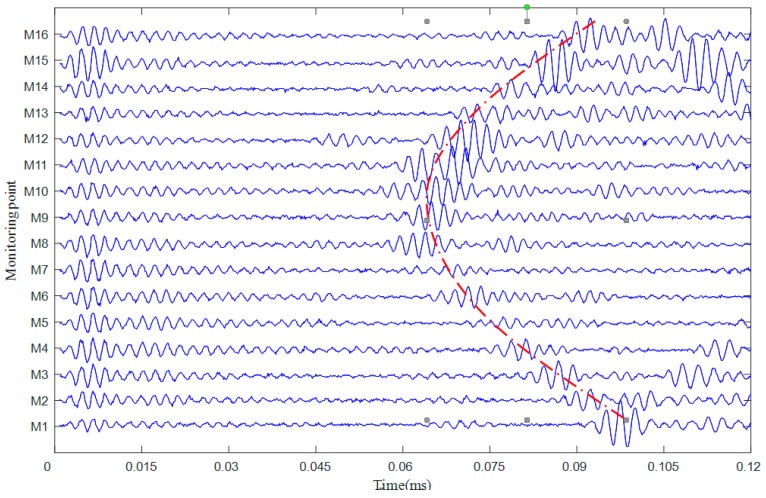
The monitoring signal excited at sensor E9.

The flight time measurement experiment of the ultrasonic guided wave inside the pipe bend was carried out using the ultrasonic guided wave experiment platform shown in Figure 7. The excitation signal was tone burst modified by a Hanning window at 200 kHz central frequency. A total of 25 × 25 = 256 waveforms were collected during this experiment. Figure 11 shows the time domain waveform of the received guided wave signal when the excitation sensor was E9, while the dotted red line is the flight time calculated by the FMM algorithm.

### 4.2. Pipe Elbow Defect Identification

**Figure 12:**
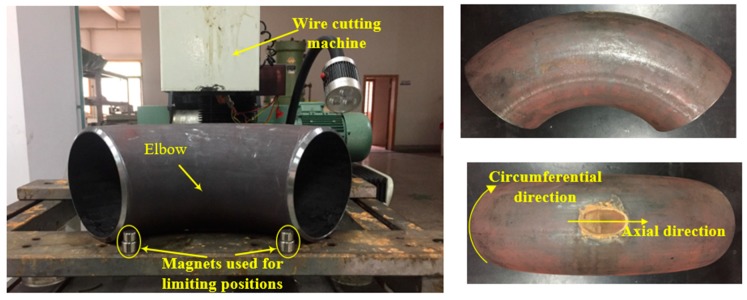
Process defect for elbow.

**Figure 13:**
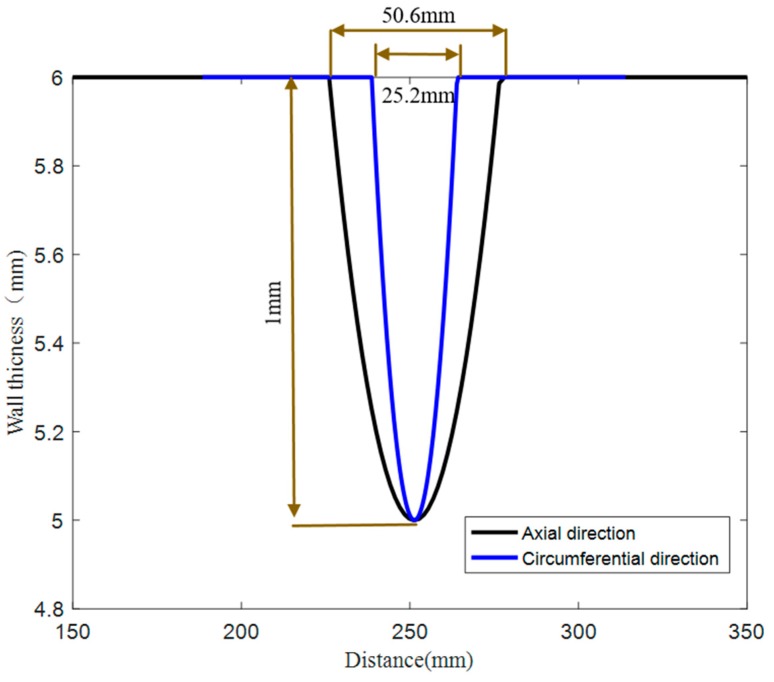
Quantitative curve of defect.

First, the defects were processed in the outer arc of the pipe elbow by the linear cutting method, as shown in Figure 12. The sizes of the defects are shown in Figure 13.

**Figure 14:**
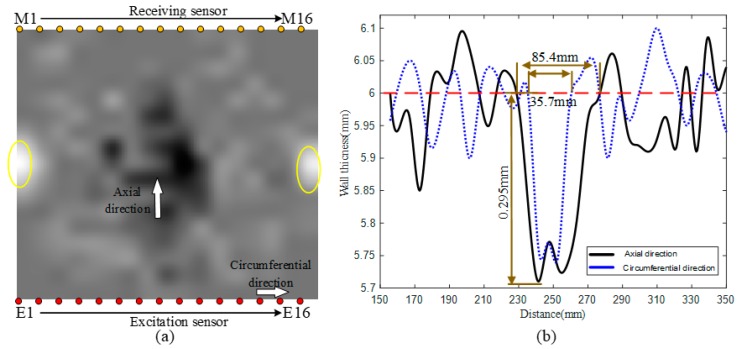
Image reconstruction results of elbow with defect in extrados: (**a**) Reconstruction results of wall thickness map; and (**b**) identification results of the defect size.

The ultrasonic guided wave detection test platform was used to conduct cross-hole scanning of the processed elbow with defects in extrados. Transmitted guided wave signals were collected, and defects were identified using the proposed sparse inversion image reconstruction technology. The sound field reconstruction results are shown in Figure 14. As shown in Figure 14a, there were velocity distortion areas caused by defects in extrados. The wall thickness of the elbow was mapped according to the dispersion curve. The fluctuation curves of the wall thickness around the defect in axial and circumferential directions are shown in Figure 14b. In Table 2, the accuracy of locating defect of the proposed method was compared with that of the method that locates defect by reflecting wave packets in signals which were generated by exciting a ring of transducers at the same time. The quantitative identification results of the defect was shown in Table 3.

(1) From Figure 14a, the velocity distortion area in the reconstructed sound field was distinguished by the proposed sparse inversion image reconstruction technology. Additionally, from Table 2, the location accuracy of the proposed method is obviously improved compared with that of the traditional method that locates defect by reflecting wave packets both in both axial and circumferential directions.

More importantly, we can see from Table 3 that the sparse inversion image reconstruction method can describe 3-D size of the defect to some extent.

**Figure 15:**
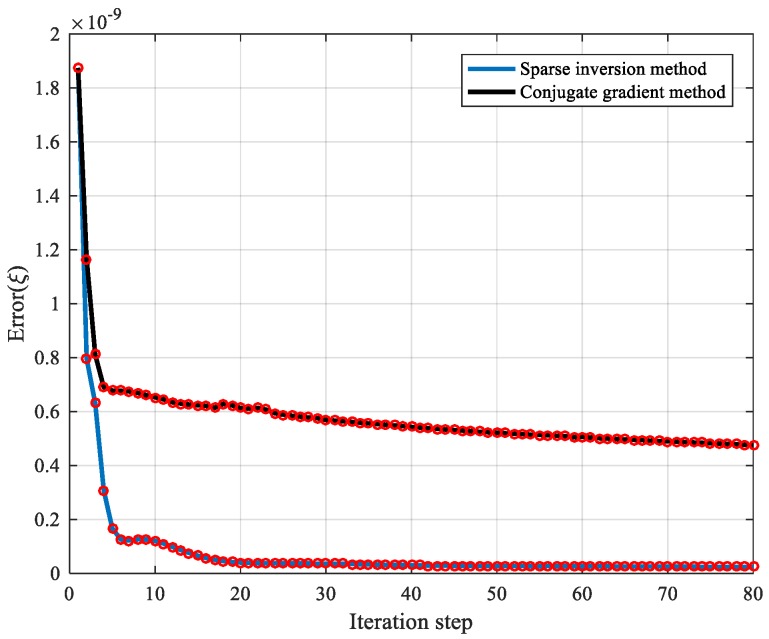
Inversion algorithm calculation process.

(2) Compared with the traditional algorithm, from Figure 15, the convergence speed of the sparse inversion method was faster, and the inversion efficiency was improved approximately 60%. The error level of the sparse inversion algorithm was approximately two orders of magnitude lower than that of the ordinary iteration. With the iteration step increase, the error evaluation value gradually decreased, and the image reconstruction of the anisotropic sound velocity field was stable and convergent.

## 5. Conclusions

This paper presented a new technique to locate the defects in elbows with higher efficiency. The technique involved building a 2-D forward modeling of ultrasonic guided wave tomography and then established the mapping relationship between the physical model and the guided wave flight time by FMM ray tracing method. Finally, the map of the elbow wall thickness was reconstructed by the sparse inversion method to identify the defects of the elbow.

In this paper, the 2-D forward acoustic model was built by unwrapping the elbows to a plate with the assumption that the wall thickness of the pipe was small relative to its diameter to improve the calculation accuracy of the flight time during the process of the forward model solution. For this purpose, starting from the Eikonal equation, the second-order difference operator was used to perform higher-order approximation of the travel time gradient operator.

The sparse inversion method was proposed to improve the efficiency of the algorithm for reconstructing the wall thickness map of the elbow based on the sparsity characteristics of the defect distribution. By substituting the velocity change factor for velocity to participate in the inversion operation, the threshold of the sound velocity change was set in combination with the dispersion curve and defect resolution, which greatly reduced the number of sound field points to be solved. The sparse inversion method was tested with simulation and experiment. Simulation results showed that the sparse inversion method can retain more sound field features because of the introduction of sound field threshold. Further experimental verification was performed at the ultrasonic guided wave detection experimental platform where the elbow was instrumented with two sensor arrays, each consisting 16 piezoelectric sensors. Experimental results showed that the location accuracy of the proposed method is obviously improved. More importantly, compared with traditional method that locates defect by reflecting wave packets, the sparse inversion image reconstruction method can describe 3-D size of the defect to some extent.

The technique presented in the paper can provide a robust technology for the identification of elbow defects in the oil and gas industries, which need a detection method with long detection distance and high efficiency.

## Figures and Tables

**Table 1:** Parameters of sound field model.

Model Size m×m	Background Velocity m/s	A1 Velocity m/s	A1 Size m×m	A2 Velocity m/s	A2 Size m×m
10×10	4000	4500	2.5×2.5	3500	2.5×2.5

**Table 2:** Location identification result of the defect.

Defect	Real Location (mm)	Identified Location (mm)	Location Accuracy	Location Accuracy of Traditional Method
Axial direction.	251.3	245.3	97.6%	94.2%
Circumferential direction	251.3	249.2	99.2%	93.7%

**Table 3:** Quantitative identification results of the defect.

Defect	Real Size (mm)	Identified Size (mm)	Identification Accuracy
$length_{\max}$	50.6	85.4	59.3%
$width_{\max}$	25.2	35.7	70.6%
$deep_{\max}$	1	0.295	29.5%
